# Cost effectiveness of intermittent screening followed by treatment versus intermittent preventive treatment during pregnancy in West Africa: analysis and modelling of results from a non-inferiority trial

**DOI:** 10.1186/s12936-016-1539-4

**Published:** 2016-09-23

**Authors:** Silke Fernandes, Elisa Sicuri, Diawara Halimatou, James Akazili, Kalifa Boiang, Daniel Chandramohan, Sheikh Coulibaly, Sory Ibrahim Diawara, Kassoum Kayentao, Feiko ter Kuile, Pascal Magnussen, Harry Tagbor, John Williams, Arouna Woukeu, Matthew Cairns, Brian Greenwood, Kara Hanson

**Affiliations:** 1 London School of Hygiene and Tropical Medicine, Keppel Street, London, WC1E 7HT UK; 2 ISGlobal, Barcelona Ctr. Int. Health Res. (CRESIB), Hospital Clínic-Universitat de Barcelona, Barcelona, Spain; 3 Department of Epidemiology of Parasitic Diseases, Faculty of Medicine, Pharmacy and Dentistry, Malaria Research and Training Centre, University of Sciences, Techniques and Technologies of Bamako, Bamako, Mali; 4 Navrongo Health Research Centre, Navrongo, Ghana; 5 Medical Research Council Unit, Fajara, Gambia; 6 Faculty of Health Sciences, University of Ouagadougou, Ouagadougou, Burkina Faso; 7 Liverpool School of Tropical Medicine, Liverpool, UK; 8 Institute of International Health, Immunology and Microbiology, Centre for Medical Parasitology and Institute of Veterinary Disease Biology, University of Copenhagen, Copenhagen, Denmark; 9 School of Medicine, University of Health and Allied Sciences, Ho, Ghana

## Abstract

**Background:**

Emergence of high-grade sulfadoxine-pyrimethamine (SP) resistance in parts of Africa has led to growing concerns about the efficacy of intermittent preventive treatment of malaria during pregnancy (IPTp) with SP. The incremental cost-effectiveness of intermittent screening and treatment (ISTp) with artemether-lumefantrine (AL) as an alternative strategy to IPTp-SP was estimated followed by a simulation of the effects on cost-effectiveness of decreasing efficacy of IPTp-SP due to SP resistance. The analysis was based on results from a multi-centre, non-inferiority trial conducted in West Africa.

**Methods:**

A decision tree model was analysed from a health provider perspective. Model parameters for all trial countries with appropriate ranges and distributions were used in a probabilistic sensitivity analysis. Simulations were performed in hypothetical cohorts of 1000 pregnant women who received either ISTp-AL or IPTp-SP. In addition a cost-consequences analysis was conducted. Trial estimates were used to calculate disability-adjusted-life-years (DALYs) for low birth weight and severe/moderate anaemia (both shown to be non-inferior for ISTp-AL) and clinical malaria (inferior for ISTp-AL). Cost estimates were obtained from observational studies, health facility costings and public procurement databases. Results were calculated as incremental cost per DALY averted. Finally, the cost-effectiveness changes with decreasing SP efficacy were explored by simulation.

**Results:**

Relative to IPTp-SP, delivering ISTp-AL to 1000 pregnant women cost US$ 4966.25 more (95 % CI US$ 3703.53; 6376.83) and led to a small excess of 28.36 DALYs (95 % CI −75.78; 134.18), with LBW contributing 81.3 % of this difference. The incremental cost-effectiveness ratio was −175.12 (95 % CI −1166.29; 1267.71) US$/DALY averted. Simulations show that cost-effectiveness of ISTp-AL increases as the efficacy of IPTp-SP decreases, though the specific threshold at which ISTp-AL becomes cost-effective depends on assumptions about the contribution of bed nets to malaria control, bed net coverage and the willingness-to-pay threshold used.

**Conclusions:**

At SP efficacy levels currently observed in the trial settings it would not be cost-effective to switch from IPTp-SP to ISTp-AL, mainly due to the substantially higher costs of ISTp-AL and limited difference in outcomes. The modelling results indicate thresholds below which IPT-SP efficacy must fall for ISTp-AL to become a cost-effective option for the prevention of malaria in pregnancy.

**Electronic supplementary material:**

The online version of this article (doi:10.1186/s12936-016-1539-4) contains supplementary material, which is available to authorized users.

## Background

Malaria in pregnancy (MiP) is associated with poor health outcomes in the mother and child, primarily during the first and second pregnancies. The most notable adverse health outcomes in moderate or high transmission settings include maternal anaemia, perinatal mortality and low birth weight (LBW) [1].

The approach to MiP prevention currently recommended by WHO consists of long-lasting insecticide treated bed nets (LLIN) and provision of sulfadoxine-pyrimethamine (SP) as intermittent preventive treatment (IPTp-SP) at each scheduled antenatal care (ANC) visit from the beginning of the second trimester until delivery [2, 3]. By clearing existing infections caused by drug-sensitive parasites as well as preventing incident infections, IPTp-SP reduces the risk of maternal anaemia, LBW and neonatal mortality [4]. Currently, 39 countries in malaria endemic sub-Saharan Africa have an IPTp policy [5]. However, analysis of national survey data from 27 countries from 2009 to 2011 estimated that despite high ANC coverage (≥2 visits, 75.1 %), only 21.5 % of the total births at risk of malaria were born to mothers who received IPTp-SP [6].

Over the last decade, the emergence and spread of high-level parasite resistance to SP in eastern and southern Africa has led to growing concerns about the effectiveness of IPTp-SP [7–10], although the loss of efficacy may be lower in pregnant women than in children under five years of age [11]. SP resistance occurs through point mutations in the genes encoding the target enzymes of SP, dihydropteroate synthase (*dhps*) and dihydrofolate reductase (*dhfr*) with prevalence of different mutant alleles varying throughout Africa. In areas with >90 % prevalence of the quintuple mutation, named after its its molecular maker “K540E”, there was continued beneficial impact of IPTp-SP on birthweight and maternal haemoglobin levels. However, IPTp-SP effectiveness becomes threatened in areas where prevalence of the sextuple *Pfdhps*-A581G mutation exceeds 10 %, such as northern Tanzania, western Kenya and southern Uganda [9, 10, 12–15]. For such areas there is a criticial need to evaluate alternative drugs to replace SP or alternative strategies using a diagnostic based test-and-treat intervention to replace IPTp. Trials of mefloquine and azithromycin with chloroquine showed that these alternatives were not sufficiently well tolerated or efficacious in pregnant women to be used for IPTp [16, 17]. IPTp with dihydroartemisinin/piperaquine has recently shown more encouraging results in Kenya and Uganda [13, 18], but there are concerns about using artemisinin combinations for widespread prophylaxis. Intermittent screening of women at each ANC visit with a rapid diagnostic test for malaria (RDT) and treatment of those with a positive result with an artemisin-based combination therapy (ACT) (ISTp) is a potential alternative approach to IPTp-SP in areas where the latter is no longer effective. A pilot study of this approach conducted in Ghana showed that ISTp was non-inferior to IPTp-SP in preventing LBW and maternal anaemia [19]. To confirm this result a non-inferiority trial which compared ISTp with artemether-lumefantrine (AL) versus IPTp-SP was conducted in four countries in West Africa (Burkina Faso, Ghana, Mali and The Gambia) [20]. The trial found ISTp-AL to be non-inferior to IPTp-SP with respect to all the primary outcomes, which were risk of LBW, mean maternal haemoglobin concentration prior to delivery and the prevalence of placental malaria [20]. However, the incidence of clinical attacks of malaria was significantly higher in women who received ISTp-AL than in those who received IPTp-SP.

Policymakers need to know the costs and cost-effectiveness of ISTp-AL and IPTp-SP as well as their clinical impact in order to inform any decisions about a change of strategy in the management of MiP. It was anticipated that the cost of ISTp-AL would be higher than IPTp-SP because (i) every woman receives an RDT (which itself is slightly more expensive than SP), (ii) it requires a more expensive drug to be administered to women screened as positive for malaria parasites, and iii) the procedure is more time-consuming. ISTp-ALis likely to be less effective where IPTp SP works well because RDTs have a low sensitivity [13] and women derive prophylactic benefit from IPTp-SP even if they are uninfected at the time of drug administration. For these reasons, the objective of this analysis was to estimate the incremental cost effectiveness of ISTp-AL versus IPTp-SP and then to simulate the effects on cost effectiveness of changes in SP efficacy due to spread of resistance. The cost effectiveness model incorporated the primary and key secondary endpoints published by Tagbor et al. [20]. In addition a cost-consequences analysis (CCA) was conducted in order to: (1) identify and consider effects that cannot easily be translated into health outcomes and (2) to report outcomes and costs separately, instead of aggregating them into a composite measure such as DALYs or QALYs and incremental cost-effectiveness ratios (ICERs) [21].

## Methods

### Trial setting, population, procedures and analysis

The trial (ClinicalTrials.gov reference: NCT01084213) enrolled a total of 5354 women in their first or second pregnancy at five sites in Burkina Faso, Ghana, Mali and The Gambia, with seasonal moderate or high malaria transmission and a prevalence of the K540E haplotype <1 % [14, 20]. All participants received an LLIN at enrolment, and then received either IPTp-SP or ISTp-AL at ANC contacts during their pregnancies. The primary objective of the trial was to establish whether ISTp-AL was non-inferior to, or in other words not worse by a clinically important amount than IPTp-SP in terms of preventing the following primary outcomes: (1) LBW (non-inferiority odds ratio (OR) margin <1.263), (2) maternal haemoglobin (Hb) concentration prior to delivery (non-inferiority margin <0.2 g/dl Hb concentration reduction) and (3) the prevalence of placental malaria (non-inferiority OR margin <1.286) [22]. More details on the trial population, procedures and data analysis are provided in Tagbor et al. [20].

### Outcomes

For the analysis described below, model outcomes were selected from the outcome categories affecting the mother or the offspring based on (1) clinical and economic importance, and (2) availability of disability weights to calculate disability-adjusted-life-years (DALYs). Based on these criteria, LBW, moderate to severe anaemia prior to delivery (both primary trial outcomes for which ISTp-AL was shown to be non-inferior) and clinical malaria (defined as an unscheduled clinic visit due to illness with a positive blood smear,- a secondary trial outcome, for which ISTp-AL was shown to be inferior to IPTp-SP) were included as model outcomes in the cost-effectiveness analysis as well as in the subsequent resistance modelling. The total DALYs in each arm were calculated by summing the DALYs from the three outcomes, as disability weights of concurring events (i.e. malaria infection and anaemia) are unknown. In addition, adverse events measured in the trial as well as intangible outcomes, such as the value of treating according to a test result rather than presumptively, were included in a cost-consequences analysis.

### Costs

The incremental fixed and variable cost to the health provider of delivering the interventions including and excluding the costs arising from the consequences of MiP (i.e. clinical malaria disease, moderate/severe anaemia and LBW) was calculated. Household costs were excluded since both interventions would be part of routine ANC visits, provided free of charge in most settings in sub-Saharan Africa and, therefore, not expected to differ between the interventions.

Economic costs were calculated and expressed in constant 2012 US$, using the local consumer price index [23] and average 2012 exchange rates [24]. Cost data collection comprised (1) health facility costing studies in the Ségou region, Mali (N = 4) and in the Upper East Region, Ghana (N = 4) and (2) two observational studies of the time nurses took to administer ISTp and IPTp in Ghana (N_ISTp_ = 18; N_IPTp_ = 20) and Malawi (N_ISTp_ = 30; N_IPTp_ = 18), conducted by the Economics Working Group of the Malaria in Pregnancy Consortium. All cost data collection was approved by the Ethics Committee of the London School of Hygiene and Tropical Medicine and by the relevant ethics committees in the countries where cost data were collected. Informed consent was obtained from each participant being observed. For more details on cost data collection methods see Additional file 1: Appendix S1.

### Analysis and model

**Fig. 1:**
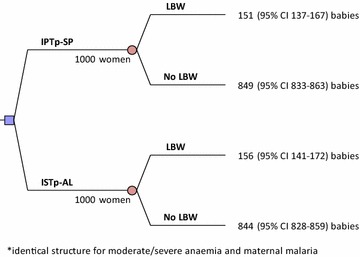
The decision tree. The decision tree model illustrates the example for LBW. All *numbers* represent the results published in Tagbor et al. [20]. The same structure was used for moderate/severe anaemia and clinical malaria. *IPTp*-*SP* intermittent preventive treatment with sulfadoxine-pyrimethamine; *ISTp*-*AL* intermittent screening and if positive followed by treatment with arthemether-lumefantrine; *LBW* low birth weight

The cost-effectiveness analysis was conducted from the health provider perspective. To account for the effects of a potential increase in SP resistance, the cost effectiveness of ISTp-AL versus IPTp-SP for decreasing efficacy of SP was simulated. In addition, the effects of reducing the costs of RDTs and AL by one half were analysed. Separate, but structurally identical decision tree models were developed for each outcome (see Fig. 1 for LBW). A lifetime horizon was adopted to show the lifelong (discounted) mortality effects of the consequences of MiP.

DALYs were estimated using disability weights from the Global Burden of Disease Study (GBD) 2010 and 2004 [25, 26], applying local life expectancies, no age weighting, and discounting at 3 %. For more details on the calculation of DALYs, see Additional file 1: Appendix S1 [25, 26].

The incremental cost-effectiveness ratio (ICER) was calculated for a hypothetical cohort of 1000 women by dividing the incremental cost of the intervention by the incremental DALYs averted [(Costs_ISTp-AL_-Costs_IPTp-SP_)—Cost from health consequences from MiP/(DALY_IPTp-SPp_-DALY_ISTp-AL_)].

The CCA separated costs and consequences into four categories, calculated per 1000 women where applicable: (i) costs, (ii) measurable outcomes contributing to DALYs, (iii) measurable outcomes that do not contribute to DALYs, and (iv) non measurable outcomes, such as the value of not giving medicines to all pregnant women. The costs were calculated including and excluding the costs associated with the health consequences, and presented with 95 % confidence intervals based on percentiles.

To illustrate the uncertainty of all estimates simultaneously we conducted a probabilistic sensitivity analysis (PSA) with 10,000 iterations, producing a point estimate and 95 % confidence interval based on percentiles for the differences in costs and effects, and an average ICER. Cost-effectiveness guidelines were used to assign an appropriate distribution to each parameter [27] (Table 1). The PSA results were plotted on the cost effectiveness plane and assessed against three frequently applied policymaker willingness-to-pay (WTP) thresholds, referred to as low (US$ 39.72), middle (US$ 238.33) and high (US$ 861.33), respectively. The low and middle threshold are the historic WHO thresholds of US$ 25 and US$ 150 adjusted for inflation [28], while the high threshold is the unweighted mean GDP per capita calculated across the four countries [29]. No deterministic sensitivity analysis was conducted, as only unrealistically large changes to a single parameter could lead to a conclusion-changing base case ICER.

**Table 1:** Input variables for the cost consequence analysis and the base case and probabilistic cost-effectiveness analysis

Parameter	Base case	Low	High	Distribution for PSA	Source
Cost estimates					
*Health care worker time cost*					
Time nurses take to provide 1 dose IPTp-SP in Ghana (min) 95 % CI (N = 18)	18.00	15.54	20.46	Gamma	Observational study of trial participants: (Ghana)
Time nurses take to provide 1 dose IPTp-SP in Malawi (min) 95 % CI (N = 18)	3.55	2.58	4.52	Gamma	Observational study of trial participants: (Malawi)
Time nurses take to provide 1 admin ISTp-AL in Ghana (min) 95 % CI (N = 3) if RDT neg	13.67	9.87	17.46	Gamma	Observational study of trial participants: (Ghana)
Time nurses take to provide 1 admin ISTp-AL in Ghana (min) 95 % CI (N = 13) if RDT pos	24.29	21.43	27.16	Gamma	Observational study of trial participants: (Ghana)
Time nurses take to provide 1 admin ISTp-AL in Malawi (min) 95 % CI (N = 22) if RDT neg	5.67	4.91	6.44	Gamma	Observational study of trial participants: (Malawi)
Time nurses take to provide 1 admin ISTp-AL in Malawi (min) 95 % CI (N = 8) if RDT pos	12.65	11.52	13.78	Gamma	Observational study of trial participants: (Malawi)
Number of administrations in IPTp-SP arm	2.06	–	–	Point estimate	[20]
Number of administrations in ISTp-AL arm	2.76	–	–	Point estimate	[20]
% of administrations of ISTp with a positive RDT result	24.7 %			Point estimate	[20]
Nurses’ monthly cost of labour, 95 % CI (US$ 2012)	346.33	164.83	527.84	Gamma	Countries MoH^a^
*Drug costs*					
Average SP price per administration, 95 % CI (US$ 2012)	0.20	0.16	0.27	Lognormal	International procurement databases^b^
Average AL price per administration, 95 % CI (US$ 2012)	2.39	1.71	3.06	Lognormal	International procurement databases^b^
Average RDT price per administration, 95 % CI (US$ 2012)	0.81	0.58	0.90	Lognormal	International procurement databases^b^
*Costs from consequences*					
Incremental days in hospital comparing LBW versus NBW (days)	0.64	0.40	0.89	Normal	Trial post partum follow up data
Cost per paediatric IP day (excluding medical supplies) (US$ 2012)	63.46	31.73	95.19	Gamma	Health facility costings (Ghana, Mali)
Cost per OP visit (excluding medical supplies) (US$ 2012)	11.76	8.45	15.06	Gamma	Health facility costings (Ghana, Mali)
Cost per IP day (excluding medical supplies) (US$ 2012)	35.25	17.63	52.88	Gamma	Health facility costings (Ghana, Mali)
Daly calculations					
Discount rate r	0.03	0.00	0.05	Point estimate	Assumption
Average age (years)^c^	20.40	–	–	Point estimate	[20]
Life expectancy women aged 20–24 years	50.12	45.11	55.13	Lognormal	GBD study 2010^d^ [32]
Life expectancy at birth	61.56	56.86	66.31	Lognormal	GBD study 2010^d^ [32]
Length disability—malaria during pregnancy (3.5 days, range 2–6)	0.010	0.005	0.016	Gamma	Assumption
Length disability—malaria related anaemia (21 days, range 14–42)	0.06	0.04	0.12	Gamma	Price et al. [33]
Length disability—LBW (years)	57.96	52.91	64.80	Lognormal	GBD 2010 study [32]
Disability weight infectious disease severe acute episode (95 % CI)	0.21	0.14	0.30	Lognormal	GBD 2010 study [25]
Disability weight maternal anaemia: moderate (95 % CI)	0.06	0.04	0.09	Lognormal	GBD 2010 study [25]
Disability weight LBW	0.11	–	–	Point estimate	GBD 2004 update (data from 1990) [26]
Mortality estimates					
LBW attributable neonatal mortality risk %	6.93	4.36	9.50	Beta	Marchant et al. [34]
CFR malaria during pregnancy %	0.0033	0.0026	0.0045	Beta	Sicuri et al. [35]
CFR moderate/severe anaemia in pregnancy %	0.01	–	–	Beta	Brabin et al. [36]
Measures of effect (trial conducted in Primi- and secundigravidae only)					
*Low birth weight*					
LBW risk IPTp-SP arm per 1000 women	151.2	136.8	166.8	Beta	[20]
LBW risk ISTp-AL arm per 1000 women	155.8	141.3	171.5	Beta	[20]
*Moderate/severe maternal anaemia (<8, 7, or 6* *g/dl)*					
Moderate/severe anaemia risk at last ANC visit IPTp-SP arm per 1000 women	16.9	11.6	24.7	Beta	[20]
Moderate/severe anaemia risk at last ANC visit ISTp-AL arm per 1000 women	23.1	16.8	31.7	Beta	[20]
*Episodes of clinical malaria*					
0 episode clinical malaria IPTp-SP arm per 1000 women	932.41	922.26	941.33	Dirichlet	[20]
1 episode clinical malaria IPTp-SP arm per 1000 women	61.99	53.45	71.78	Dirichlet	[20]
2 episodes clinical malaria IPTp-SP arm per 1000 women	5.6	3.38	9.27	Dirichlet	[20]
3 episodes clinical malaria IPTp-SP arm per 1000 women	0.4	0.36	0.44	Dirichlet	[20]
0 episode clinical malaria ISTp-AL arm per 1000 women	899.85	887.88	910.67	Dirichlet	[20]
1 episode clinical malaria ISTp-AL arm per 1000 women	84.08	74.14	95.21	Dirichlet	[20]
2 episodes clinical malaria ISTp-AL arm per 1000 women	14.95	10.98	20.32	Dirichlet	[20]
3 episodes clinical malaria ISTp-AL arm per 1000 women	0.37	0.05	2.65	Dirichlet	[20]
Measures of effect from cochrane review used for modelling of decreasing SP efficacy in IPTp arm					
Relative risk of LBW comparing IPTp-SP versus none or placebo	0.81	0.67	0.99	n/a	[30]
Relative risk of sev/mod anaemia comparing IPTp-SP versus none or placebo	0.60	0.47	0.75	n/a	[30]
Relative risk of antenatal parasitaemia comparing IPTp-SP versus none or placebo	0.38	0.24	0.59	n/a	[30]

Parameters are shown for all countries where the clinical trial was conducted in [32] (Burkina Faso, Ghana, Mali and The Gambia)

*ANC* antenatal care; *CFR* case fatality rate; *DALY* disability adjusted life years; *HCW* health care worker; *IPTp*-*SP* intermittent preventive treatment with sulfadoxine-pyrimethamine; *ISTp*-*AL* intermittent screening and if positive followed by treatment with arthemether-lumefantrine; *LBW* low birth weight; *MoH* Ministry of Health; *95* *% CI* 95 % confidence interval

^a^Salary scale and an average allowance package for nurses from Ministry of Health in Burkina Faso, Ghana, Mali and The Gambia for 2012

^b^Cost for dose of SP, AL and per RDT was calculated accounting for 5 % wastage, 10 % insurance and freight and 10 % in country transport

^c^Average age was used from the trial

^d^The life expectancy was analysed from the Global burden of disease 2010 database for the subgroups of interest (i.e. female only, age 20–24, trial countries and both gender, at birth, trial countries respectively)

The impact of different levels of SP resistance on the ICER was subsequently modelled, by changing the efficacy of IPTp-SP while holding the efficacy of ISTp-AL constant as measured in the trial. The measured risk of the model outcomes (LBW, severe/moderate anaemia and clinical malaria) in the IPTp-SP arm of the trial was assumed to be the maximum possible efficacy and labelled as 100 %. All trial participants were assumed to have slept under an LLIN. Details of how SP resistance affects IPTp-SP efficacy are not clearly understood, therefore we assumed a linear relationship between decreasing IPTp-SP efficacy and outcomes. Data from the Cochrane review by Radeva-Petrova et al. [30] (comparing IPTp-SP with placebo or no intervention) were used to calculate the extrapolated risk of the model outcomes in the IPTp-SP group by percentage point change in efficacy, from 100 % (maximum) to 0 % (minimum). As the Cochrane review authors were unable to stratify IPTp efficacy by bed net use, the effect of bed nets on these modelled outcomes was explored in three scenarios, by assuming: (1) bed nets have no effect and the full extrapolated risk was attributable to IPTp-SP (used as the starting point for simulation only), (2) bed nets bear 1/2 and (3) 2/3 of the burden and, therefore, only 1/2, or 1/3, respectively, of the extrapolated risk of the model outcomes was attributable to IPTp-SP. Subsequently, the incremental costs, incremental DALYs and ICERs comparing ISTp-AL with IPTp-SP at each percentage point of decreasing SP efficacy for each of the three bed net scenarios were calculated.

Finally, these hypothetical results were used to calculate the threshold level of SP efficacy at which ISTp-AL would become cost effective, stratified by WTP and assumption about the contribution of bed nets. For more details on how the resistance modelling was conducted, please refer to Additional file 1: Appendix S1. All resistance simulations included the costs arising from health consequences of MiP and at bed net coverage levels estimated in the most recent Demographic and Health Surveys in the four trial countries (unweighted average of 47.7 % across the four countries).

Analysis of international procurement databases indicated an overall downwards trend in the unit cost of RDTs and AL from 2010 to 2014. Therefore, the impact of reducing these commodity costs by 50 % (with the standard error held constant) on the ICER and in the resistance analysis was explored.

To be able to show simultaneously how the cost effectiveness changes by level of SP efficacy, assumption of the contribution of bed nets and bed net coverage, we used a net monetary benefit (NMB) function instead of the ICER, because (a) the simulated results cannot easily be depicted when the denominator of a ratio approaches 0 and (b) because NMB includes the WTP threshold in the formula. The formula for NMB is as follows: NMB = (∆DALY averted x WTP)-∆Costs. When using NMB, an intervention becomes cost effective when the NMB is ≥0.

Stata (version 12, StataCorp, College Station, Texas) was used to analyse the observational data whilst the international procurement data, the health facility costs and the decision tree model were analysed in Excel (Microsoft Office 2013), the latter also using Visual Basic for Applications.

## Results

### Consequences

Per 1000 women there were 151 (95 % CI 137;167) vs 156 (95 % CI 141;172) LBW babies, 17 (95 % CI 12;25) vs 23 (95 % CI 17;32) cases of severe or moderate anaemia and 74 (95 % CI 61;92) vs 115 (95 % CI 96;144) episodes of clinical malaria at unscheduled visits in the IPTp-SP and ISTp-AL groups, respectively (Table 3). The total burden was estimated as 755.1 and 782.9 DALYs per 1000 women in the IPTp-SP and ISTp-AL arms, respectively (Table 3).

### Costs

Table 2 shows the itemized cost results. The total cost (including health worker time and cost of supplies) per administration of IPTp-SP was US$ 0.79 (95 % CI 0.53; 1.12). If screening was negative, the cost per administration of ISTp-AL was US$ 1.45 (95 % CI 1.12; 1.85) and US$ 4.06 (95 % CI 3.29, 4.97) if screening was positive (see Table 2).

**Table 2:** Itemized cost results

Type of cost	Cost parameter	Cost in US$ 2012, mean (95 % CI)
Intervention cost	Average SP price per administration^a^	0.2 (0.16–0.25)
	Average AL price per administration^a^	2.39 (1.71–3.06)
	Average RDT price per administration^a^	0.81 (0.58–0.90)
	HCW time cost per dose of IPTp-SP	0.59 (0.33–0.91)
	HCW time cost per administration of ISTp-AL if screened negative	0.64 (0.36–1.00)
	HCW time cost per administration of ISTp-AL if screened positive	0.84 (0.47–1.31)
	Total cost per administration of IPTp-SP (HCW time and commodity cost)	0.79 (0.53–1.12)
	Total cost per administration of ISTp-AL if screened negative (HCW time and commodity cost)	1.45 (1.12–1.85)
	Total cost per administration of ISTp-AL if screened positive (HCW time and commodity cost)	4.06 (3.29–4.97)
Health provider costs excluding medical supplies	Cost per OP visit	11.78 (9.18–14.68)
	Cost per IP day adult	35.27 (19.99–55.07)
	Cost per paediatric IP day (nursery)	63.51 (36.3–99.27)
Health provider costs of consequences	Total average short term cost per LBW baby	40.93 (19.52–71.19)
	Total average cost per moderate/severe anaemia case	10.15 (6.76–15.17)
	Total average cost per clinical malaria case	13.71 (6.12–24.65)

*AL* arthemether-lumefantrine; *HCW* health care worker; *IP* inpatient; *IPTp*-*SP* intermittent preventive treatment with sulfadoxine-pyrimethamine; *ISTp*-*AL* intermittent screening and if positive followed by treatment with artemether-lumefantrine; *LBW* low birth weight; *OP* outpatient; *SP* sulfadoxine-pyrimethamine

^a^Including freight, insurance, wastage and in country transport

The cost per outpatient visit for malaria treatment was US$ 11.78 (95 % CI 9.18; 14.68), per adult inpatient day US$ 35.27 (95 % CI 19.99; 55.07) and per paediatric inpatient day US$ 63.51 (95 % CI 36.30; 99.27), the latter two excluding medical supplies. The final cost of health consequences of MiP was estimated to be US$ 40.93 (95 % CI 19.52; 71.19) for the short-term consequences of LBW, US$ 10.15 (95 % CI 6.76; 15.17) for a moderate/severe anaemia case and US$ 13.71 (95 % CI 6.12; 24.65) for a clinical malaria case during pregnancy.

Table 3 shows the costs of administering the interventions to 1000 pregnant women, which amounted to US$ 1631.84 (95 % CI 1100.11; 2316.97) for IPTp-SP and US$ 5778.77 (95 % CI 4701.65; 7039.15) for ISTp-AL, when excluding the costs arising from health consequences of MiP. After including costs arising from health consequences it rose to US$ 9006.54 (95 % CI 5610.30; 13,680.56) for IPTp-SP and US$ 13,972.79 (95 % CI 10,199.65; 18,983.86) for ISTp-AL (Table 3). In summary, if 24.7 % of women screened in the ISTp-AL arm have a positive RDT test result, as measured in the trial, and if the costs of the consequences of MiP are included, ISTp-AL costs on average around US$5 more per woman per pregnancy than IPTp-SP. If 10 % (as in The Gambia) or 50 % (as in Ghana) of the RDT screenings were positive the costs per woman would amount to US$ 3.88 and US$ 6.73, respectively.

**Table 3:** Cost consequence analysis

		IPTp-SP	ISTp-AL
*Costs* per 1000 pregnant women	Health provider excluding consequences^b^ (US$ 2012)	1631.84 (1100.11–2316.97)	5778.77 (4701.65–7039.15)^a^
	Health provider including consequences^b^ (US$ 2012)	9006.54 (5610.30–13,680.56)	13,972.79 (10,199.65–18,983.86)^a^
*Measurable outcomes* contributing to DALYs per 1000 women [20]	LBW	151 (137–167)	156 (141–172)
	severe/moderate anaemia	17 (12–25)	23 (17–32)
	clinical malaria (total episodes)^c^	74 (61–92)	115 (96–144)
*Other measurable* outcomes per 1000 women and side effects [20, 37]	Placental malaria: active infection (acute and chronic)	245 (224–265)	242 (222–262)
	Dizziness	46 (38–54)	25 (19–31)
	Sleeplessness	13 (9–17)	7 (4–10)
	Weakness	30 (24–37)	18 (13–23)
	Nausea	22 (16–28)	13 (9–17)
	Vomiting	45 (37–53)	26 (20–32)
*Non measurable* outcomes	Intrinsic value of giving *less drugs* to pregnant women		
	Value to *individual* of *knowing* about positive test		
	Value of *knowing* about positive test for *surveillance*		
	Deprivation of treatment for *false negatives*		

*DALY* disability adjusted life years; *IPTp*-*SP* intermittent preventive treatment with sulfadoxine-pyrimethamine; *ISTp*-*AL* intermittent screening followed by treatment with artemether-lumefantrine; *LBW* low birth weight

^a^At 24.7 % of administration where RDT showed a positive result

^b^Costs from consequences = ∑ short term costs LBW baby; costs mod/sev anaemia episode; costs MiP episode

^c^Clinical malaria defined as illness + positive slide at an unscheduled visit

### Cost effectiveness analysis

Compared with IPTp-SP, delivering ISTp-AL to 1000 pregnant women led to an excess of 27.8 DALYs, of which 81.3 % were attributable to LBW, 5.9 % to severe/moderate anaemia and 12.8 % to clinical malaria. With an incremental cost of US$ 4929.0 per 1000 women, this produces an ICER of US$ −177.1/DALY averted (Table 4).

**Table 4:** Cost effectiveness analysis

Deterministic results (base case)	
DALYs LBW averted	−22.64
DALYs mod/sev anaemia averted	−1.63
DALYs MiP averted	−3.56
Total DALY IPTp-SP	755.1
Total DALY ISTp-AL	782.93
Total cost IPTp-SP (US$ 2012)^a^	9037.84
Total cost ISTp-AL (US$ 2012)^a^	13,966.84
Δ Costs (US$ 2012)^a^	4929.00
Δ DALYs	−27.83
*ICER (US$/DALY)* ^a^	−*177.10*
Probabilistic results	
DALYs LBW averted	−23.15 (−128.35 to 80.71)
DALYs mod/sev anaemia averted	−1.63 (−4.35 to 0.94)
DALYs MiP averted	−3.58 (−5.69 to 1.83)
Total DALY IPTp-SP	753.55 (631.07 to 895.88)
Total DALY ISTp-AL	781.91 (657.92 to 928.64)
Total cost IPTp-SP (US$ 2012)^a^	9006.54 (5610.30 to 13,680.56)
Total cost ISTp-AL (US$ 2012)^a^	13,972.79 (10,199.65 to 18,983.86)
Δ Costs (US$ 2012)^a^	4966.25 (3703.53 to 6376.83)
Δ DALYs	−28.36 (−134.18 to 75.78)
*Average ICER (US$/DALY)* ^a^	−*175.12*

^a^Including the costs from health consequences caused by malaria during pregnancy

**Fig. 2:**
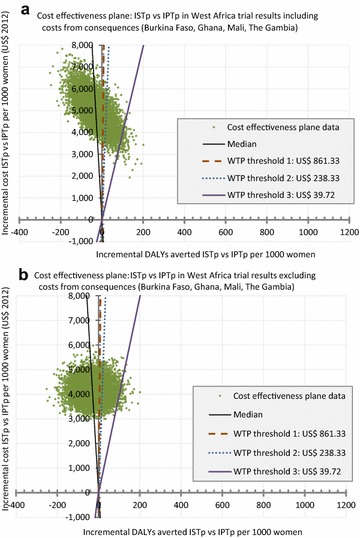
Cost effectiveness planes: the *graphs* display the results of two different Monte Carlo simulations with 10’000 iterations each using the value ranges and distributions specified in Table 1. The *different coloured lines* illustrate the median and three willingness-to-pay (WTP) thresholds. WTP threshold 1 of US$ 861.33 is the GDP/capita averaged over the four countries. WTP threshold 2 and 3 represent the original thresholds defined by WHO in 1993 as highly attractive and attractive, both inflated to US$ 2012. **a** shows the results including the costs occurring from the consequences of malaria during pregnancy and **b** excluding these costs

The negative ICER is not driven by cost savings, but rather by a slightly lower (not statistically significant) point estimate of efficacy in the ISTp-AL group. The results of the PSA including the costs of the health consequences of MiP are shown in Table 4 and Fig. 2a. Per 1000 women the difference in cost amounted to US$ 4966.25 (95 % CI US$ 3703.53; 6376.83) more for ISTp-AL with an excess of 28.36 (95 % CI −75.78; 134.18) DALYs. The average ICER from the PSA was −175.12 (95 % CI −1166.29; 1267.71) US$/DALY averted. The results excluding the costs of health consequences of MiP are plotted in Fig. 2b yielding a difference in costs of US$ 4146.93 (95 % CI 3420.66; 4978.48) per 1000 women resulting in an ICER of −146.29 (95 % CI −1039.11; 1123.15) US$/DALY averted. The less widely dispersed simulation points on the CE plane reflect less uncertainty around the cost estimate when costs of the health consequences of MiP are excluded.

### Cost-consequences analysis

Table 3 shows the results of the cost-consequences analysis. Consequences that could not be included in the calculation of DALYs included placental malaria, a primary outcome of the trial, and side effects measured in the trial, all expressed as cases per 1000 women. All side effects which occurred at statistically significantly different rates in the ISTp-AL and IPTp-SP arms are shown. Of the measured side effects there were fewer cases of dizziness, sleeplessness, weakness, nausea and vomiting reported in the ISTp-AL arm. In addition to these measurable differences, there were also other non-measurable outcomes that should be considered. These include benefits, such as the intrinsic value of giving fewer drugs to pregnant women and only administering drugs to women who are infected; the value to women of knowing their infection status; and the value to society of identifying malaria infection, particularly for surveillance purposes. There are also potential deleterious effects, such as failure to treat women with a false negative test.

### Modelling of cost effectiveness for decreasing efficacy of SP and decreased commodity costs

**Fig. 3:**
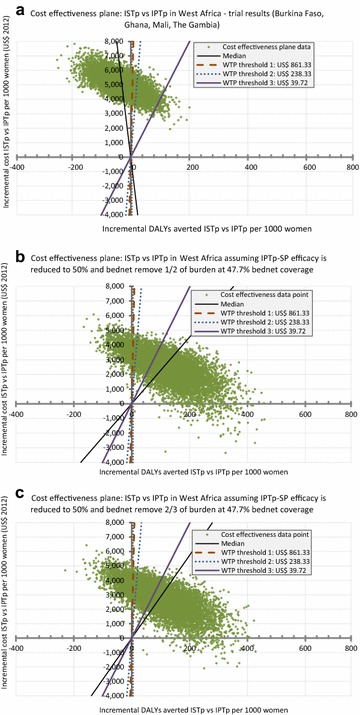
Simulation results: cost effectiveness plane at SP efficacy level of 50 % with a bed net coverage of 47.7 % [37] and with costs from consequences included. **a** shows the trial results replicated from Fig. 2a for comparison purpose, **b**, **c** show the simulation results for the assumption that bed nets bear 1/2 and 2/3 respectively, of the predicted burden of LBW, severe/moderate anaemia and clinical malaria

**Fig. 4:**
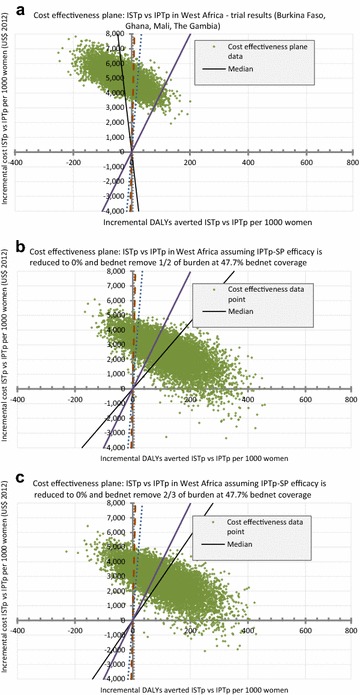
Simulation results: cost effectiveness plane at SP efficacy level of 0 % with a bed net coverage of 47.7 % [37] and with costs from consequences included. **a** shows the trial results replicated from Fig. 2a for comparison purpose, **b**, **c** show the simulation results for the assumption that bed nets bear 1/2 and 2/3 respectively, of the predicted burden of LBW, severe/moderate anaemia and clinical malaria

Figures 3 and 4 shows the simulation results for a hypothetical reduction of SP efficacy to 50 and 0 % respectively, of the current level. Figures 3a and 4a replicate Fig. 2a, while Figs. 3b, c and 4b, c show the simulation results assuming that bed nets bear 1/2 or 2/3 of the extrapolated outcome burden when SP efficacy is reduced. Simulation results assuming no contribution of bed nets can be found in the Additional file 1: Appendix S1. By reducing SP efficacy levels from 100 % to 50 % or 0 %, more simulation points lie in the North Eastern (top right) quadrant of the cost effectiveness plane and to the right of the WTP threshold (the criterion for being cost-effective with respect to this threshold).

Table 5 (in column 2 and 3) indicates the efficacy levels at which ISTp-AL becomes cost effective stratified by WTP threshold and the assumptions concerning burden prevented by bed net use. For example at the middle WTP threshold ISTp-AL becomes cost-effective when the efficacy of IPTp-SP is between (i) 63–64 % or (ii) 59–60 % of current SP efficacy levels, assuming that bed nets bear (i) 1/2 or (i) 2/3 of the extrapolated outcome burden. For the highest and lowest WTP threshold this range lies between 69–73 % and 12–20 %, respectively.

**Table 5:** Threshold modelling for decreasing SP efficacy: Efficacy levels of IPTp-SP at which ISTp-AL becomes cost effective stratified by (1) two assumptions made on burden prevented by bed net use at a bed net coverage of 47.7 % and (2) willingness to pay threshold

	Full costs of RDT and AL		Costs of RDT and AL halved	
	Bed nets bear 1/2 of extrapolated burden	Bed nets bear 2/3 of extrapolated burden	Bed nets bear 1/2 of extrapolated burden	Bed nets bear 2/3 of extrapolated burden
WTP 1 = US$ 861.33/DALY averted	Between 72 and 73 %	Between 69 and 70 %	Between 74 and 75 %	Between 71 and 72 %
WTP 2 = US$ 238.33/DALY averted	Between 63 and 64 %	Between 59 and 60 %	Between 68 and 69 %	Between 65 and 66 %
WTP3 = US$ 39.72/DALY averted	Between 19 and 20 %	Between 12 and 13 %	Between 41 and 42 %	Between 35 and 36 %

Results are shown for the full costs of RDT and AL as well as for the costs reduced by 50 %

The first column shows the three willingness to pay (WTP) thresholds

The first row indicates if the full costs for RDT and AL were used or if they were halved

The second row of the table show the two assumptions made regarding burden prevented by bed net use with bed nets bearing 1/2 and 2/3 respectively of the extrapolated burden of LBW, severe/moderate anemia and clinical malaria when IPTp-SP efficacy decreases Results shown here are based on a 47.7 % bed net coverage level

Additional results summarizing the effects of three key variables on cost effectiveness: (i) the level of IPTp-SP efficacy, ranging from 0 to 90 % of current efficacy, (ii) bed net coverage and (iii) the WTP threshold are shown in the Additional file 1: Appendix S1.

Finally, reducing the costs of ACT and RDT by 50 % leads to an ICER of US$ −107.7/DALY averted (∆Cost = US$ 2998.1, ∆ DALY = −27.8). Table 5 (in column 4 and 5) shows the results of the threshold analysis when the costs of the ACT and RDT were halved. The effect of reducing commodity costs leads to a higher threshold of SP efficacy at which ISTp-AL becomes cost effective, in other words, with lower commodity costs, SP needs to lose less of its efficacy for ISTp-AL to become cost-effective.

## Discussion

At current levels of cost and efficacy, it is not cost-effective to switch from IPT-SP to IST-AL in areas where *Plasmodium falciparum* remains sensitive to SP. This is because IST-AL is more costly than IPTp-SP and no more effective at reducing the adverse effects of malaria during pregnancy. However, it is possible to use a cost-effectiveness model to simulate how cost-effectiveness would change with the loss of efficacy of IPTp-SP resulting from a potential development of SP resistance, and with a reduction in commodity costs. The threshold at which IST-AL becomes cost effective compared with IPTp has been assumed to vary depending on the contribution of bed nets to reducing the burden of MiP; another key variable is decision maker WTP to avert a DALY. The analysis presented here shows that for the highest WTP threshold ($861 per DALY averted), ISTp-AL becomes cost-effective at levels of IPTp efficacy ranging from 69 to 79 % of current levels. At a threshold of $238/DALY averted, a greater loss of IPTp-SP efficacy is needed for ISTp-AL to become cost-effective, and for the lowest threshold ($40/DALY averted), IPTp-SP efficacy must fall to 12–36 % of current levels before IST-AL is more cost-effective. These thresholds are higher when commodity costs are halved, i.e. this level is found between 71 and 80 % for the highest and 35–54 % for the lowest WTP threshold.

The cost-consequences analysis represents an effort to introduce into the decision-making metrics the effects of important consequences that cannot be readily incorporated into the calculation of DALYs. For example, as malaria incidence diminishes, the justification for presumptive treatment will need to be re-examined, however safe the drug used for preventive treatment. Valuations of this benefit could be elicited from women and communities. However, integrating this benefit into outcome measures may require moving to a cost-benefit or impact evaluation framework. Similarly, the value of information on infection, either to women individually or as a contribution to surveillance in a context of malaria elimination, could be examined empirically. On the other hand there is also the possible negative impact of women who falsely test negative, who could potentially be deprived of essential treatment. However, for the purpose of this analysis the authors have elected to enumerate, rather than to quantify, these benefits or disadvantages.

This analysis is subject to a number of important limitations. First, the Cochrane review [30] includes results from more recent trials, including some conducted in areas where some level of SP resistance is expected, biasing downwards the efficacy of IPT-SP prior to the simulated decrease due to resistance. Second, the presence of bed nets introduces a series of complications related to distinguishing the incremental effect of IPTp-SP when bed nets are already addressing some share of the burden of MiP. The Cochrane review efficacy estimates were not stratified by bed net use, therefore assumptions about the incremental effectiveness of IPTp-SP in addition to bed nets were needed. All trial participants were given a bed net but actual adherence to bed nets was not measured and, therefore, it was not possible to stratify efficacy by bed net use, although it is expected to have been high under trial conditions.

The costs of ISTp-AL were affected by the proportion of women who test positive when screened with an RDT, which in turn is dependent on malaria transmission intensity. However, even at the lowest observed level of positive test results, ISTp-AL was not cost-effective as the cost of the test itself exceeds that of IPTp-SP.

Costs also depend on the number of times a woman receives each intervention during a pregnancy. At the time of the trial, the policy in three of the trial countries was for women to receive two doses of IPTp-SP, resulting as measured in the trial in 2.06 administrations per woman in the IPTp-SP and 2.76 in the ISTp-AL arm. Although it would be feasible to explore how the frequency of administration would affect costs, it is impossible to estimate how this would influence efficacy, therefore trial estimates were used. As an indication, increasing the number of administrations of IPTp-SP to 2.76 per woman reduced the difference in costs between the two interventions to US$ 4.4 per woman. Transmission level is also likely to influence cost-effectiveness. More evidence is needed from low transmission settings, which also partially overlap with sextuple mutant settings as to how this balance will work out.

This model has focused on simulating how cost effectiveness of ISTp-AL changes if SP efficacy decreases. It is currently not known how SP resistance levels translate into IPTp efficacy and ISTp has yet to be shown to be more effective in a high resistance setting. One trial conducted in Western Kenya, where the prevalence of the sextuple A581G mutant was measured at 5.8 %, found ISTp with dihydroartemisinin–piperaquine (DP) to be inferior to IPTp-SP [13]. It is thought that in settings with even higher prevalence of the A581G sextuple mutant, IPTp-SP has almost no efficacy in preventing clinical malaria, MiP attributable LBW and anaemia [14, 15], however the loss of SP efficacy in such settings remains to be studied. Naidoo and Roper suggested that most *P. falciparum* resistance patterns appear first in East Africa and usually spread to Central and West Africa within 15 years [31].

Decisions about a change in strategy to prevent MiP will be based on many factors in addition to effectiveness and cost-effectiveness. Feasibility and acceptability to mothers and health workers will be important, as will be the feasibility of policy change. In addition, total cost and affordability will be crucial.

## Conclusion

At the current level of SP efficacy and transmission intensity found in this trial in West Africa, switching from IPTp-SP to ISTp-AL is not recommended as ISTp-AL is not more effective and costs considerably more per woman. However, as our modelling suggests, in settings with >10 % prevalence of the sextuple mutant, where IPTp has reduced or no efficacy, ISTp-AL has the potential to be a viable and cost-effective option. Any continued decrease in commodity costs would positively affect this transition.

## Additional file

10.1186/s12936-016-1539-4 The additional file contains further detail on the methods used in the analysis and additional results.

## Abbreviations

AL: artemether-lumefantrine
ACT: artemisin-based combination therapy
ANC: antenatal care
CCA: cost consequence analysis
CEA: cost effectiveness analysis
DALY: disability adjusted life year
Hb: haemoglobin
HCW: health care worker
ICER: incremental cost effectiveness ratio
IPTp: intermittent preventive treatment of malaria during pregnancy
ISTp: intermittent screening for malaria during pregnancy and if positive followed by treatment
GBD: global burden of disease
LBW: low birth weight
LLIN: long-lasting insecticide-treated net
MiP: malaria during pregnancy
NMB: net monetary benefit
PSA: probabilistic sensitivity analysis
RDT: rapid diagnostic test
SP: sulfadoxine-pyrimethamine
WHO: World Health Organization
WTP: willingness to pay
