# Supplementary material for: Cost effectiveness of intermittent screening followed by treatment versus intermittent preventive treatment during pregnancy in West Africa: analysis and modelling of results from a non-inferiority trial
Source: Malar J. 2016 Sep 23;15:493. doi: 10.1186/s12936-016-1539-4 (PMC5035479; doi:10.1186/s12936-016-1539-4)
Supplement: Supplementary file 1 — 10.1186/s12936-016-1539-4 The additional file contains further detail on the methods used in the analysis and additional results. [file 12936_2016_1539_MOESM1_ESM.docx]

Additional file 1: Appendix S1

**Cost effectiveness of Intermittent Screening followed by Treatment versus Intermittent Preventive Treatment during pregnancy in West Africa: analysis and modelling of results from a non-inferiority trial**

Silke Fernandes, MSc^1*^; Elisa Sicuri, PhD^2^; Diawara Halimatou, MD^3^; James Akazili, PhD^4^; Kalifa Boiang, PhD^5^; Daniel Chandramohan, PhD^1^; Sheikh Coulibaly, PhD^6^; Sory Ibrahim Diawara, MD^3^; Kassoum Kayentao, PhD^3,7^; Feiko ter Kuile, PhD^7^; Pascal Magnussen, MD^8^; Harry Tagbor, DrPH^1^; John Williams, MB^4^; Arouna Woukeu, PhD^1^; Matthew Cairns, PhD^1^; Brian Greenwood, MD^1^ ; Kara Hanson, ScD^1^

Appendix S1:

S_Methods

- The cost of administering the interventions
- The cost of health consequences arising from Malaria during pregnancy (MiP)
- Estimating Disability adjusted life years (DALYs)
- Modelling of cost-effectiveness when IPTp-SP efficacy is decreasing

S_Results

- Additional results summarizing the effects of three key variables on cost effectiveness using Net monetary benefit

S_References

S_Methods:

The cost of administering the interventions included the cost of nurses’ time and the cost of drugs and supplies required to administer IPTp-SP or ISTp-AL. National salary scales and average allowance packages for different grades of nurses and midwives in the public sector were averaged across trial countries and used to calculate the average monthly cost of a nurse. The value of nurses’ time was estimated by multiplying the cost per minute of nurses’ time by the time required to administer IPTp or ISTp measured in the two observational studies. The costs of RDTs (for ISTp), drugs and other supplies were calculated by analyzing transaction data from international procurement databases [1-3], as applicable , adjusting non-sequentially for wastage (5%), insurance & freight (10%) and internal transport (10%). The number of doses of AL provided to women in the ISTp arm was estimated from the proportion of women who screened positive for malaria at each visit (24.7%, 95% CI .23.7%-25.7%).

**The costs of the health consequences arising from MiP** were calculated for clinical malaria, moderate/severe anaemia and the short term costs of LBW, using the ingredients approach for medical supplies combined with a step down costing for all other capital and recurrent costs[4]. The ingredients approach starts with the inputs used to produce a medical procedure or treatment and places a monetary value on each. The step down method allows an appropriate proportion of the clinical staff and overhead costs to be allocated to the output of interest. [4]. Table S1 presents details on the assumptions made when calculating the costs of the three health outcomes. The short term costs of LBW were estimated by multiplying the average cost per day in a paediatric ward (excluding medical supplies) by the number of additional days a LBW baby was admitted in hospital after delivery compared with a non-LBW baby, estimated using data collected as part of the clinical trial. Medical supply costs for management of LBW babies were excluded because LBW can result in a variety of complications or none.

**Estimating DALYs:** To calculate DALYs, disability weights from the 2010 Global Burden of Disease Study (GBD) were used where available, otherwise we resorted to 2004 GBD study estimates.[5, 6] DALYS were discounted at 3% (essentially deducting 3% per year to reflect the lower value of DALYs incurred in the future) and no age weighting. We used average life expectancy at birth (averaged over the four countries across males and females) for LBW and at 20-24 years of age (averaged over the four countries for females only) to estimate years of life lost due to moderate/severe anaemia and clinical malaria in mothers [5]. For LBW an attributable neonatal mortality risk of 6.9% (95% CI 4.4-9.5%) as reported by Marchant *et al.* for East Africa was applied [7], which is comparable to the median attributable neonatal mortality risk for babies born prematurely at appropriate weight for gestational age in Africa (55.8/ 1000 live births) reported by the Child Health Epidemiology Group [8]. The case fatality rate used for malaria during pregnancy was 0.33% [9] and for moderate/severe anaemia 1.0% [10]. The length of disability for clinical malaria during pregnancy was assumed to be 3.5 days (range 2-6), 21 days for malaria related anaemia (range 14-42) [10] and lifelong for LBW.

**Modelling of cost-effectiveness when IPTp-SP efficacy is decreasing**

1) Cochrane review data: To estimate the hypothetical efficacy of IPTp versus no intervention at different levels of SP efficacy, the relative risks of LBW (0.81, 95% CI 0.67-0.99), severe/moderate anaemia (0.60, 95% CI 0.47-0.75) and antenatal parasitaemia (0.38, 95% CI 0.24-0.59) comparing IPTp-SP versus placebo or no intervention from the Cochrane review were used as model inputs. Then, for each outcome the hypothetical change in the relative risk arising from a decrease in SP efficacy was calculated in 1 percentage point steps, beginning with the estimate reported in the review, until no further effect was observed (i.e. relative risk = 1.0). The standard error of all three measures of effect was kept constant to calculate hypothetical 95% confidence intervals.

2) Use of Cochrane review estimates to calculate hypothetical risks in IPTp-SP arm: To model the changing risks of LBW, severe/ moderate anaemia and clinical malaria as IPTp efficacy declines, the hypothetical relative risks calculated in Step 1 were used to estimate the extrapolated risks of LBW, severe/moderate anaemia and clinical malaria in the IPTp-SP arm of the trial at different levels of decreasing SP efficacy (on a scale of 100% to 0%). The measured risk in the IPTp-SP arm of the trial was assumed to be the maximum possible efficacy and labelled as 100%. This was varied in 1% steps using the hypothetical relative risks until no effect of IPTp-SP was reached, labelled as 0%.

3) Resistance analysis: To understand the level of SP efficacy below which IPTp-SP would no longer be more cost-effective than ISTp-AL, the hypothetical risks in the IPTp-SP arm calculated in Step 2 (on a scale of 100% to 0%) were used to calculate incremental DALYs, incremental costs and the ICER comparing ISTp-AL with IPTp-SP for each percentage point of decreasing SP efficacy. Finally these hypothetical results were used to calculate the threshold level of SP efficacy at which ISTp-AL would become cost effective, stratified by WTP.

In the control of malaria in pregnancy the effects of IPTp and ITNs can be described as complementary and it has been shown that the simultaneous use of both interventions provides optimal protection against LBW. As the Cochrane review did not stratify IPTp efficacy by bednet use, we explored the effect of bednets on the extrapolated risk of the outcomes of interest in three scenarios, by assuming: 1) by using the actual Cochrane review efficacy estimates which do not stratify by bednet use, essentially assuming that the presence of bednets would not prevent any of the “additional” burden (of LBW, severe/moderate anaemia and clinical malaria) and the full extrapolated risk was attributable to IPTp-SP; 2) by assuming that bednet use prevents half and 3) 2/3 of the extrapolated risk, essentially assuming that only 1/2, or 1/3 respectively, of the extrapolated risk was attributable to IPTp-SP. The effect of reduction in SP efficacy was calculated separately for each of the three bednet scenarios. Scenario 1 uses an unrealistically extreme assumption and is used only as a starting point for the simulation. Scenarios 2) and 3) are consistent with different studies exploring the impact of bednets, IPTp-SP and the combination of both on maternal and neonatal outcomes [11, 12].

**S_Results**

**Additional results summarizing the effects of three key variables on cost effectiveness using Net monetary benefit**:

Add Base case graphs!!!

Figures S1a)-c) summarize the effects of three key variables on cost effectiveness: i) the level of IPTp-SP efficacy, ranging from 0% to 90% of current efficacy, ii) bednet coverage on the x-axis and iii) the WTP threshold, which was WTP threshold of 861.33 US$ / DALY averted for figure S1a; WTP threshold of 238.33 US$/ DALY averted for figure S1b) and WTP threshold of 39.72 US$/DALY averted for S1c). All results displayed in figure S1a) to S1c) are based on the assumption that as SP efficacy decreases, the extrapolated burden of LBW, severe/moderate anaemia and clinical malaria for women taking IPTp-SP is only1/2 of the maximum predicted increase due to the presence of bednets.. The y-axis manipulates the ICER into a net monetary benefit (NMB) of ISTp-AL versus IPTp-SP according to the following formula: **NMB= (∆DALY averted x WTP)-∆Costs**, implying an intervention to be cost-effective when NBW ≥0. We drew a line at NMB of =0 to indicate the SP efficacy level (shown as % of current efficacy) above which ISTp-AL versus IPTp-SP would become cost-effective which varies by WTP threshold, but also by bednet coverage. The dotted lines in the graph are plots of the NMB (y-axis) by bednet coverage (x-axis) for different levels of SP efficacy displayed in 10% steps with 90% SP efficacy shown in light blue at the bottom and 0% efficacy in light brown at the top. For example in graph S1a) it is shown that at bednet coverage of 60%, ISTp-AL compared to IPTp-SP becomes cost-effective at around 70% of current SP efficacy if we assume that bednets bear 1/2 of the predicted outcome burden and a WTP threshold of 861.33 US$/DALY averted. However, after keeping all assumptions as above except for changing the WTP threshold to 39.72 US$/DALY averted, ISTp-AL only becomes cost-effective at approximately 14% of current SP efficacy. Similarly, the SP efficacy level at which ISTp-AL becomes cost-effective changes with bednet coverage. For example using a WTP threshold of 238.33 US$/ DALY averted, at unlikely bednet coverages of 0% and 100%, ISTp-AL becomes cost-effective at approximately 71% and 48% of current SP efficacy levels. This highlights the importance of the WTP threshold, as well as bednet coverage in addition to level of SP efficacy when assessing future cost-effectiveness of ISTp-AL in the light of decreasing SP efficacy due to resistance.

S_Tables

***Supplement***

***Table S1: Parameters and assumptions for the calculations of health facility costs per case of LBW, severe/ moderate anaemia and clinical malaria***

| Parameter | Base case | Low | High | Distribution for PSA | Source |
| --- | --- | --- | --- | --- | --- |
| LBW |  |  |  |  |  |
| Incremental days in hospital comparing LBW vs NBW | 0.64 | 0.40 | 0.89 | Normal | Trial data MA5 Post partum follow up form comparing LBW vs NBW |
| Cost per pediatric IP day excl medical supplies (US$ 2012), n=3, SD=24.19 | 63.46 | 31.73 | 95.19 | Gamma | health facility costing Ghana and Mali, SE not used, for CI: +/-50% |
| Anaemia |  |  |  |  |  |
| Cost per Hb test Hemocue (adjusted to US$ 2012 using CPI change USA) (US$ 2001: 0.647638 US$/test) | 0.84 | 0.10 | 0.84 | uniform | Lara et all., 2005, Evaluation and costs of different haemoglobin methods for use in district hospitals in Malawi |
| % of women with severe/mod anaemia seeking care receiving Hb test | 100% | 0% | 0% | point estimate | estimate |
| Cost per anaemia treatment (US$ 2012) | 0.69 | 0.58 | 0.79 | Lognormal | MSH international procurement database |
| Cost per blood transfusion unit replacement donor (16.23 US$ 2005) adjusted to US$ 2012 using CPI change USA (+/-10%) | 19.13 | 17.22 | 21.05 | Gamma | Lara et all., 2007, Laboratory costs of a hospital-based blood transfusion service in Malawi |
| Cost per blood transfusion unit centralised system (56 US$ 2005) adjusted to US$ 2012 using CPI change USA (+/-10%) | 65.81 | 59.23 | 72.39 | Gamma | Lara et all., 2007, Laboratory costs of a hospital-based blood transfusion service in Malawi |
| Average number of transfusion units in pregnancy, if transfusion required | 2 | 0 | 0 | point estimate | estimate |
| % of blood transfusion replacement donor in Africa | 78% | 75% | 80% | beta | Lara et all., 2007, Laboratory costs of a hospital-based blood transfusion service in Malawi |
| % women with moderate/ severe anaemia seeking care | 50% | 40% | 60% | beta | estimate |
| % women with moderate/ severe anaemia seeking care, who are treated in OP/ ANC | 90% | 85% | 95% | beta | estimate |
| % women with moderate/ severe anaemia seeking care who are treated in IP | 0% | 0% | 0% | dependent on variable above | estimate |
| % women with moderate/ severe anaemia in IP receiving blood transfusion | 20% | 10% | 30% | beta | estimate |
| Cost per OP visit excl medical supplies (US$ 2012), N=8; SD=3.96 | 11.76 | 8.45 | 15.06 | Gamma | health facility costing Ghana and Mali |
| Cost per IP day excl medical supplies (US$ 2012), N=2, SD = 7.86 | 35.25 | 17.62 | 52.87 | Gamma | health facility costing Ghana and Mali, SE not used, for CI +/-50%, N too small |
| Average number of days admitted for acute severe/moderate anaemia | 2 | 1 | 3 | Lognormal | expert opinion |
| MiP |  |  |  |  |  |
| Cost per MiP treatment 1st trimester (Quinine+Cindamycin and range) (US$2012) | 13.41 | 0.14 | 13.41 | Lognormal | health facility costing Ghana and Mali |
| Cost per MiP treatment 2nd/3rd trimester (Coartem and range) (US$2012) | 2.39 | 0.14 | 13.41 | Lognormal | health facility costing Ghana and Mali |
| % of women with MiP seeking care in health facility | 55% | 27% | 83% | beta | Review Hill.J. et all. , 2014, Women's access and provider practices for the case management of malaria during pregnancy: a systematic review and meta-analysis |
| % of women seeking care who receive a diagnostic test in OP | 70% | 60% | 90% | beta | estimate |
| % of women seeking care who receive a diagnostic test in IP | 95% | 90% | 100% | beta | estimate |
| % of women seeking care in 1st trimester | 15% | 10% | 20% | beta | estimate |
| % of women with MiP seeking care treated in OP/ ANC | 90% | 85% | 95% | beta | estimate |
| Average number of days admitted for acute MiP | 3.00 | 2.00 | 4.00 | Lognormal | expert opinion |

**S_Figures**

***Figure S1*:** Net monetary benefit (on y-axis) of ISTp-AL vs IPTp-SP by level of SP efficacy (90% to 0% efficacy) and bednet coverage (0% to 100% on x-axis). Bednets are assumed to prevent 1/2 of the extrapolated burden of LBW, severe anaemia and clinical malaria when efficacy of IPTp-SP is reduced through simulation from 100% to 0%. Net monetary benefit is calculated as:

NMB= (∆DALY averted x WTP)-∆Costs

NMB=Net monetary benefit

DALY=Disability adjusted life years

WTP=Willingness to pay

Different WTP thresholds were used to calculate the Net monetary benefit with figure S1a) showing results for WTP threshold=861.33 US$ / DALY averted; S1b) for WTP threshold= 238.33 US$/ DALY averted and S1c) for WTP threshold 39.72 US$/DALY averted

***Figure S2***

**Simulation results: Cost effectiveness plane at SP efficacy level of 50% (S2a) and 0% (S2b)** and with costs from consequences included. Both graphs assume that bednets do not matter when IPTp-SP efficacy decreases and therefore do not bear any of the predicted burden of LBW, severe/moderate anaemia and clinical malaria. These figures are to show the increase in burden if the simulation does not take account of bednets and are not considered relevant in practice however were the starting point for including bednets into our simulation.

**S_References**

1. **The Global Fund Procurement dashboard** [<http://bi.theglobalfund.org/analytics/saw.dll?Dashboard>]

2. **International Drug Price Indicator Guide** [<http://erc.msh.org/mainpage.cfm?file=1.0.htm&module=DMP&language=English>]

3. The Global Fund: **Affordable Medicine Facility - malaria (AFMm) - summary report on co-paid ACTs.** 2010-2013.

4. Conteh L, Walker D: **Cost and unit cost calculations using step-down accounting.** *Health Policy Plan* 2004, **19:**127-135.

5. Salomon JA, Vos T, Hogan DR, Gagnon M, Naghavi M, Mokdad A, Begum N, Shah R, Karyana M, Kosen S, et al: **Common values in assessing health outcomes from disease and injury: disability weights measurement study for the Global Burden of Disease Study 2010.** *Lancet* 2012, **380:**2129-2143.

6. **Global Burden of Disease 2004 update: disability weights for diseases and conditions** [<http://www.who.int/healthinfo/global_burden_disease/GBD2004_DisabilityWeights.pdf>]

7. Marchant T, Willey B, Katz J, Clarke S, Kariuki S, ter Kuile F, Lusingu J, Ndyomugyenyi R, Schmiegelow C, Watson-Jones D, Armstrong Schellenberg J: **Neonatal mortality risk associated with preterm birth in East Africa, adjusted by weight for gestational age: individual participant level meta-analysis.** *PLoS Med* 2012, **9:**e1001292.

8. Katz J, Lee AC, Kozuki N, Lawn JE, Cousens S, Blencowe H, Ezzati M, Bhutta ZA, Marchant T, Willey BA, et al: **Mortality risk in preterm and small-for-gestational-age infants in low-income and middle-income countries: a pooled country analysis.** *Lancet* 2013, **382:**417-425.

9. Sicuri E, Bardaji A, Nhampossa T, Maixenchs M, Nhacolo A, Nhalungo D, Alonso PL, Menendez C: **Cost-effectiveness of intermittent preventive treatment of malaria in pregnancy in southern Mozambique.** *PLoS One* 2010, **5:**e13407.

10. Brabin BJ, Hakimi M, Pelletier D: **An analysis of anemia and pregnancy-related maternal mortality.** *J Nutr* 2001, **131:**604S-614S; discussion 614S-615S.

11. Eisele TP, Larsen DA, Anglewicz PA, Keating J, Yukich J, Bennett A, Hutchinson P, Steketee RW: **Malaria prevention in pregnancy, birthweight, and neonatal mortality: a meta-analysis of 32 national cross-sectional datasets in Africa.** *Lancet Infect Dis* 2012, **12:**942-949.

12. ter Kuile FO, van Eijk AM, Filler SJ: **Effect of sulfadoxine-pyrimethamine resistance on the efficacy of intermittent preventive therapy for malaria control during pregnancy: a systematic review.** *JAMA* 2007, **297:**2603-2616.
